# Hand hygiene promotion in long-term care facilities (LTCF) – a cluster randomized controlled trial

**DOI:** 10.1186/1753-6561-5-S6-O65

**Published:** 2011-06-29

**Authors:** ML Ho, WH Seto, TS Lam, LC Wong, TY Wong

**Affiliations:** 1 Centre for Health Protection, Hong Kong, China; 2 Hospital Authority, Hong Kong, China

## Introduction / objectives

This is the 1st cluster randomized controlled trial showing effectiveness of WHO Multimodal Strategy in promoting hand hygiene (HH) among health care workers (HCW) of LTCF with only 1 registered nurse per home at one time.

## Methods

18 elderly homes with a total of 812 HCW were randomly allocated to 2 intervention arms and a control arm. The study was conducted during November 2009 to July 2010. WHO Multimodal Strategy was employed, homes under interventional arms I and II were supplied with slightly powdered gloves and powderless gloves respectively. Controls were provided with similar promotion materials unrelated to HH. Direct observation by trained nurses was used to measure HH compliance. Self-administered questionnaires served to assess HH knowledge of HCW. Disease notification data during 2007-2010 were used to calculate incidence rate ratio (IRR).

## Results

A total of 11,669 HH opportunities were observed. HH compliance increased from 27.0%to 60.6% and from 22.2%to 48.6% in intervention arms I and II respectively. Both intervention arms showed increase in HH compliance after intervention compared to controls of 21.6% (both p<0.001). “Before touching patient” among the WHO five moments for HH, activity index >40 opportunities/hour, physiotherapist/occupational therapist were associated with less improvement. Mean knowledge score increased from 5.5 to 6.6 after intervention. Respiratory outbreaks (IRR=0.12; 95% CI: 0.01-0.93; p=0.04) and MRSA admissions (IRR=0.61; 95% CI: 0.38-0.97; p=0.04) reduced after intervention.

## Conclusion

Promotion program applying WHO Multimodal Strategy is effective in improving HH among HCW in LTCF.

## Disclosure of interest

None declared.

